# Structural, Morphological, Optical and Electrical Characterization of Gahnite Ferroan Nano Composite Derived from Fly Ash Silica and ZnO Mixture

**DOI:** 10.3390/ma15041388

**Published:** 2022-02-14

**Authors:** Sushree Saraswati Panda, Hara Prasada Tripathy, Priyabrata Pattanaik, Dilip Kumar Mishra, Sushanta Kumar Kamilla, Asimananda Khandual, William Holderbaum, Richard Sherwood, Gary Hawkins, Shyam Kumar Masakapalli

**Affiliations:** 1 Semiconductor Research Laboratory, Faculty of Engineering and Technology (ITER), Siksha ’O’ Anusandhan (Deemed to be University), Bhubaneswar 751030, India; ss.sushreesaraswati@gmail.com (S.S.P.); hara345@gmail.com (H.P.T.); priyabratapattanaik@soa.ac.in (P.P.); dilipmishra@soa.ac.in (D.K.M.); sushantakamilla@soa.ac.in (S.K.K.); 2 Department of Textile Engineering, Odisha University of Technology and Research, Ghatikia, Bhubaneswar 751029, India; asimte@cet.edu.in; 3 School of Biological Science, Biomedical Engineering, University of Reading, Whiteknights RG6 6AY, UK; 4 Infrared Multilayer Laboratory, Atmospheric, Oceanic and Planetary Physics, Clarendon Laboratory, Parks Road, Oxford OX1 3PU, UK; richard.sherwood@physics.ox.ac.uk (R.S.); gary.hawkins@physics.ox.ac.uk (G.H.); 5 School of Basic Sciences, Indian Institute of Technology Mandi, Kamand, Mandi 175005, India; shyam@iitmandi.ac.in

**Keywords:** fly ash, ZnO, gahnite ferroan, band pass filter, NTCR

## Abstract

The synthesis of a high value-added product, gahnite ferroan nano composite, from a mixture of fly ash silica and ZnO is a low-cost and non-expensive technique. The XRD pattern clearly reveals the synthesized product from fly ash after leaching is a product of high-purity gahnite ferroan composite. The grains are mostly cubical in shape. The optical band gap of powdered gahnite ferroan nano composite is 3.37 eV, which acts as a UV protector. However, the bulk sample shows that the 500 to 700 nm wavelength of visible light is absorbed, and UV light is allowed to pass through. So, the bulk sample acts as a band pass filter of UV light which can be used in many optical applications for conducting UV-irradiation activity. Dielectric permittivity and dielectric loss increase with a rise in temperature. The increase in the ac conductivity at higher temperatures denotes the negative temperature coefficient resistance (NTCR) behavior of the material.

## 1. Introduction

Gahnite ferroan, (Zn,Fe)Al_2_O_4_, is a mineral present in small amounts in rock but has no bearing on the classification of the rock, similar to zircon in granite. A volcanic eruption has metamorphosed the mineral sulfide ore bodies by creating gahnite with different impurities, such as iron and magnesium [1]. This also occurs as the segregations in quartz veins and quartzo-feldspathic rock. Gahnite is present throughout the mineralized zones and thus contains pyrite, sphalerite, chalcopyrite and sulfosalts. This mineral has been found naturally in so many places [1,2,3,4,5] starting from in the Falun mine (Sweden), Wind River Mountain (Wyoming, United States), Silberberg mine (Bavaria, Germany), near Parelhas (Rio Grande do Norte, Brazil), Wodgina and Greenbushes (Western Australia), Victoria Range (Nelson, New Zealand) and the Mamandur area (Tamil Nadu, India). Due to globalization, these rare minerals are in the last stage of vanishing. Recreating this type of mineral for different applications is the prime objective of this research work, by implementing the waste management techniques to form value-added products such as gahnite ferroan.

Pulverized fuel ash (PFA), fly ash (FA) or coal combustion residuals (CCRs) are produced from coal-fired boilers with flue gases. Coal firing started in the 1920s for thermal power generation, as a result of which millions of tons of FA and related by-products have been produced worldwide over the past century [6,7]. As a result of the huge production of FA and its toxic behavior, the recycling of FA has become a primary concern in the climate of clean environmental economies. A large portion of FA has been utilized for making cement [8], but still, a huge amount of residual FA is dumped in landfills [9] and ash ponds [10]. The composition of FA and its impact on the environment [6] is instigating researchers to work extensively on the reformation of these materials for different potential applications. For several decades, FA has been utilized as concrete [11], bricks [6], geopolymer [12,13], a low-cost absorbent for the removal of organic compounds [14,15], poison gas [16], water treatment [17,18], earth filler and the synthesis of zeolite. FA has also been utilized in the agricultural sector as a fertilizer to increase the productivity of the soil, in paints and enamels, wood substitute composites, floor tiles and wall tiles [19]. A wide variety of compounds are found in the composition of FA, including silicon dioxide (SiO_2_), aluminum oxide (Al_2_O_3_) and iron oxide (Fe_2_O_3_). Elements such as manganese (Mn), zinc (Zn), nickel (Ni), lead (Pa) and arsenic (As) are found predominantly in different forms of fly ash, depending on the production method. The above traceable compounds can be further modified, and the altered end product utilized in different potential applications [20,21,22,23].

In particular, zinc oxide (ZnO) has been chosen to alter the FA from its base nature to form rare minerals. Zinc aluminate (gahnite, ZnAl2O4) is a rare mineral formed due to the alteration product of sphalerite ((Zn,Fe)S compositions). A wide energy band gap of 3.8 eV (large absorbance in UV region) and chemical stability in high-temperature environments are all useful properties for researchers to investigate. Aluminum-based spinels depict an intriguing group of oxide ceramics with diverse applications [24]. Among these, gahnite prominently serves as a pigment, in optoelectronics and as a transparent semiconductor, a photo catalyst, and a ceramic material [21,22,23,25,26,27,28,29,30]. An enormous number of applications can be possible by using gahnite as the base material. In this research article, a method is performed to synthesize gahnite ferroan by utilizing a mixture of FA and ZnO. The utilization of FA is an entirely new and novel application of this research work. Structural, morphological, optical and electrical characterizations are carried out to confirm the formation and different properties of gahnite ferroan.

## 2. Experimental Section

Fly ash (FA) with a specific gravity of 2.20 g/cm^3^ on ignition (LOI: 2.90%) was procured from the National Aluminum Company Limited (NALCO), Angul 759145, Odisha, India. The quantification of various compounds present in the fly ash was analyzed by a chemical route and is listed in Table 1.

Similarly, zinc acetate ((CH_3_COO)_2_Zn ∙ 2H_2_O) (98%), ethylene glycol (HOCH_2_CH_2_OH) (99%), glycerol (C_3_H_8_O_3_) (98%), 2-propanol (CH3CHOHCH3) (99%), triethylamine (C_2_H_5_)_3_N) (99%) and nitric acid (HNO_3_) (70%) from Emplura, Merck, were purchased commercially for the synthesis of ZnO sol-gel without further purification, and they have CAS numbers of 557-34-6, 107-21-1, 56-85-1, 67-63-0, 102-71-6 and 7697-37-2, respectively. The chemical route technique was employed to form ZnO sol-gel. For the synthesis of ZnO in sol-gel form, zinc acetate (9.74 g), ethylene glycol (25 mL) and glycerol (12 drops: 6 mL) were mixed and stirred using a magnetic stirrer with a fixed bed temperature of 130 °C, until a transparent solution without any granular particles was obtained. While cooling down to room temperature, 2-propanol (42 mL) and triethylamine (11 mL) were added in the mixture and the solution was continuously stirred for 15 min. The solution was transferred to a 500 mL beaker and placed in a sand bath with the bed temperature maintained at 200 °C [31]. In lukewarm conditions, fly ash (10 g) was added and stirred using a glass rod to remove lumps before the beginning of the pyrophoric process with the addition of triethylamine (5 mL) and nitric acid (10 mL), respectively for reaction process propagation. The continuous bed temperature led to the redox mixture leaving behind ample amounts of carbon-containing powder. The collected sample was ground and underwent calcination at 1300 °C for 5 h. The carbon-free sample was collected and underwent hydrofluoric leaching at a ratio of 1:5 with distilled water for each 2 g of carbon-free sample for 7 h. After the leaching, the sample was collected through filtratrion (Whatman filter paper). The wet filter paper was kept in an oven for 3 h at 200 °C and then in a furnace for 5 h at 600 °C. After this process, the final product was found to be 0.76 g out of 2 g of leached synthesized powder.

## 3. Characterization

X-ray diffraction patterns of the prepared sample were performed utilizing Rigaku Ultima-IV to study the crystal structure, with radiation 1.5406 Å (Cu Kα source) wavelengths on 2θ ranging from 20° to 80° at room temperature. Scanning electron microscopy (FEI company of USA (S.E.A) PTE LTD, model- Nova Nano SEM-450) and transmission electron microscopy (FEI company of USA, model- FP5022/22-Tecnai G220 S-TWIN) were carried out to study the three- and two-dimensional structures, along with the average grain size of the produced sample. The Nova Nano SEM-450 was equipped with backscattered electron and secondary electron detectors and an EDAX energy-dispersive X-ray spectrometer. Fourier transform infrared (FTIR) spectral analysis wavenumber ranging from 350 cm^−1^ to 7800 cm^−1^ were scanned utilizing a FT-IR 4600 instrument (Japan Spectroscopic Company). Transmittance, absorbance and semiconducting properties were analyzed by implementing UV-VIS-NIR spectrum analysis (Jasco V-750 spectrophotometer, Japan). The precision of the instrument was enhanced by double beam optics with a variable spectral bandwidth. The dielectric and electrical properties were analyzed by recording the AC parameters, such as capacitance (C), loss tangent (tanδ), impedance (Z), and phase (θ) using an LCR meter (NF model ZM2376, Japan).

## 4. Results and Discussion

**Figure 1:**
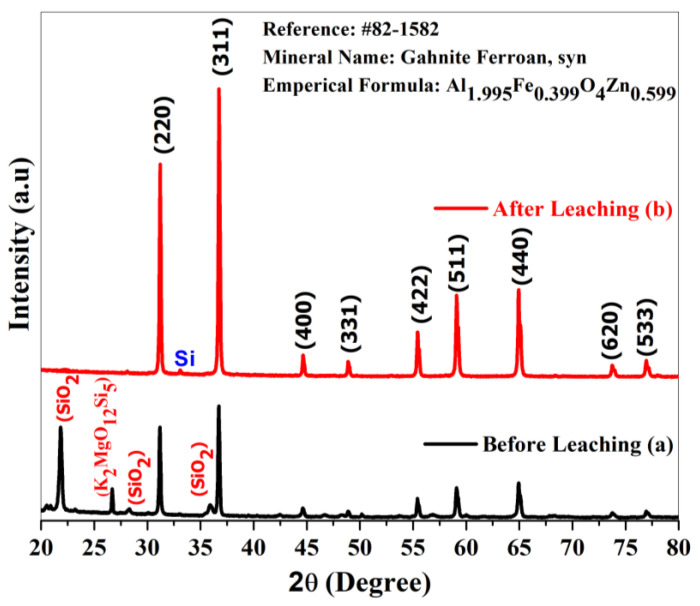
X-ray diffraction pattern of gahnite ferroan composites (**a**) before leaching and (**b**) after leaching obtained powder sample within Bragg’s diffraction angles of 20° to 80°.

In order to visualize the sample formation, X-ray diffraction pattern analysis was carried out. Figure 1 illustrates the stacked individual X-ray diffraction patterns of (a) before-leaching, and (b) after leaching, respectively. The hydrofluoric acid leaching had a prominent impact on the purity and thus is clearly visible in Figure 1a,b. After leaching, the pattern in Figure 1b shows the pure form of the produced sample. Without any baseline correction, the pattern was matched with standard database # 82-1582. This reveals the mineral name, along with the empirical formula of the formed composite. Gahnite ferroan (Al_1.995_Fe_0.339_O_4_Zn_0.599_) belongs to the cubic crystal system. From inter planar spacing, the Scherrer equation and reference #82-1582, the lattice constant, crystallite size and volume of the unit cell were calculated. The lattice constant with the unit cell volume of gahnite ferroan are well matched with the reference values reported in JCPDS # 85-1582, and the calculated values are 8.8114 Å, 532.22 Å and 8.2991 Å, 571.60 Å, respectively. The calculated average crystallite size of the gahnite ferroan sample is 40.15 nm. The observed lattice strain of the sample is 8.143 × 10^−6^, which is calculated from a Williamson–Hall plot.

**Figure 2:**
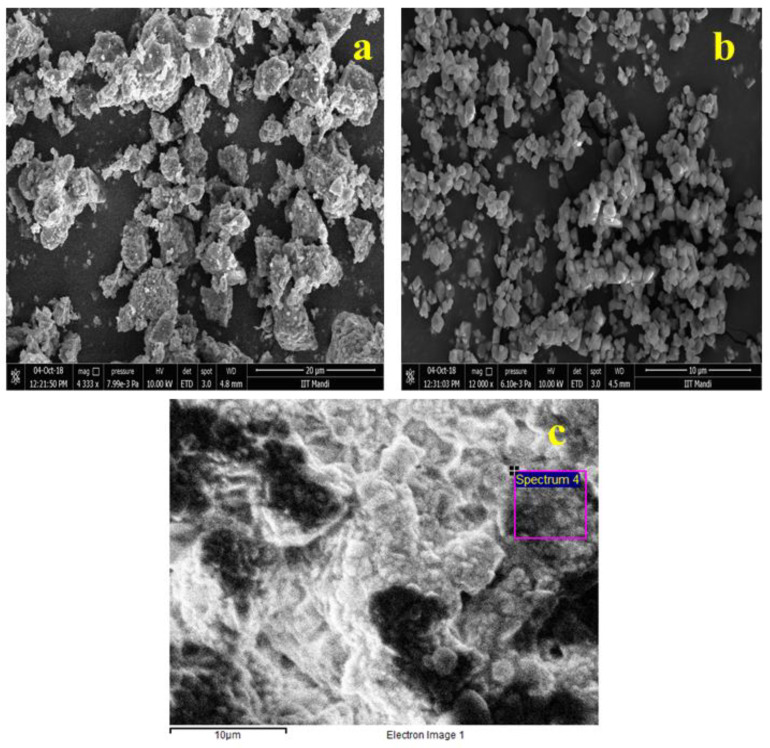
Scanning electron micrographs of gahnite ferroan composites (**a**) before leaching at 4.3 k mag and 7.99 × 10^−3^ pa, with scale size 20 µm, (**b**) after leaching, obtained powder sample at 12 k mag and 3.72 × 10^−3^ pa, with scale size 10 µm, and (**c**) bulk sample, with scale size 10 µm.

**Figure 3:**
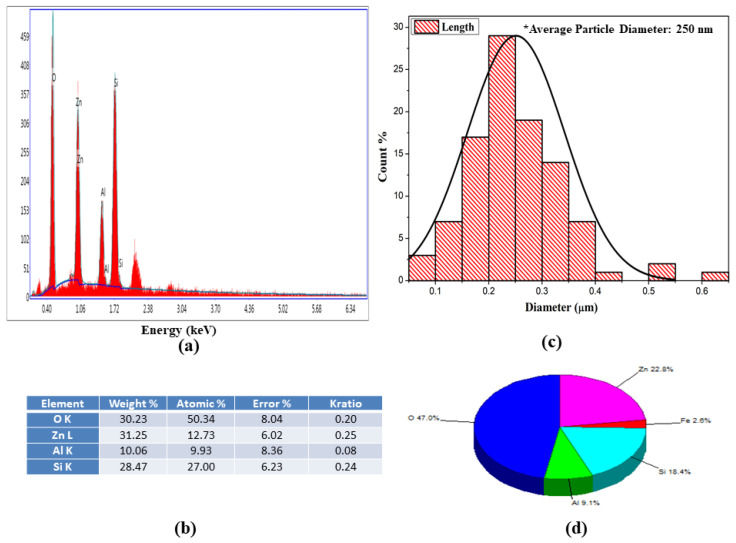
(**a**) Energy-dispersive X-ray patterns of gahnite ferroan composites; (**b**) particle size distribution of gahnite ferroan composites; (**c**) elemental analysis of gahnite ferroan composites; and (**d**) quantitative weight percentage of bulk gahnite ferroan composite.

**Figure 4:**
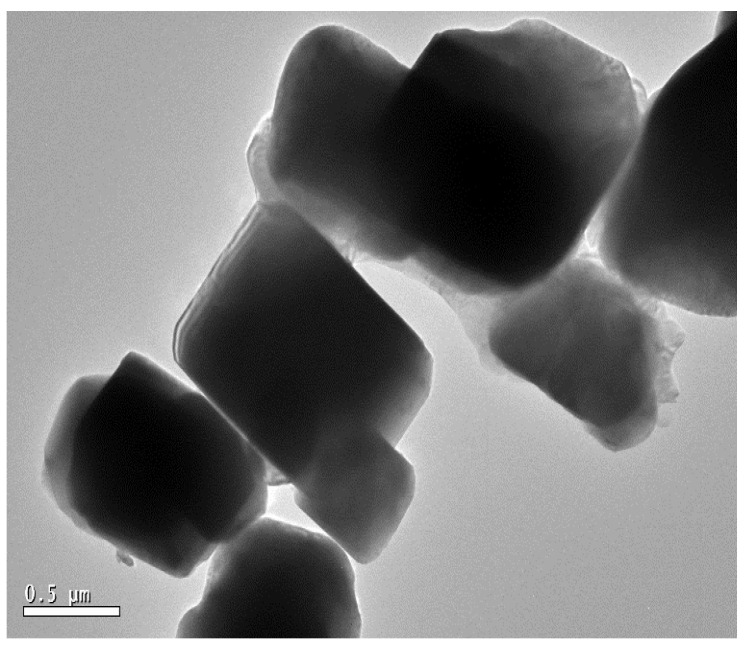
Transmission electron microscopic image of gahnite ferroan composite.

Surface morphology, structural appearance, texture, and chemical composition of the samples were visualized using scanning electron microscopy in conjunction with an EDS system. Figure 2a shows the produced sample before leaching at 4.3 k mag and 7.99 × 10^−3^ pa, with a scale size of 20 µm, Figure 2b after leaching obtained powder sample at 12 k mag and 3.72 × 10^−3^ pa, with a scale size of 10 µm, and Figure 2c bulk sample, with a scale size of 10 µm. The energy-dispersive X-ray pattern with a smart quant table depicts the material composition in or on the surface for quantitative information. The EDS pattern with the elemental smart quantization table is illustrated in Figure 3. The weight percentage values of the elements O (oxygen), Z (zinc), Al (aluminum) and Si (silicon) are 30.23, 31.25, 10.06 and 28.47, respectively. The sample also underwent transmission electron microscopy for the visualization of internal structure and particle size analysis. Figure 4 shows the TEM image of the gahnite ferroan composite. The average particle size of gahnite ferroan is 460 nm.

**Figure 5:**
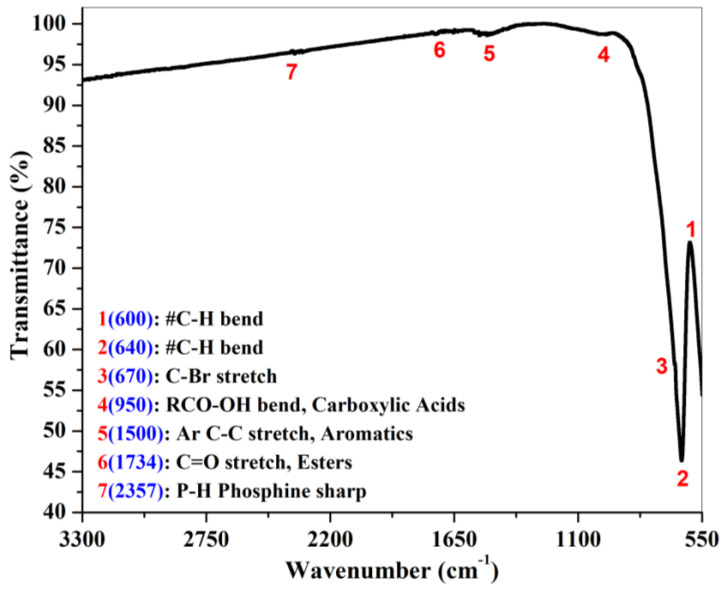
Fourier-transform infrared (FTIR) spectrum of gahnite ferroan composite.

**Figure 6:**
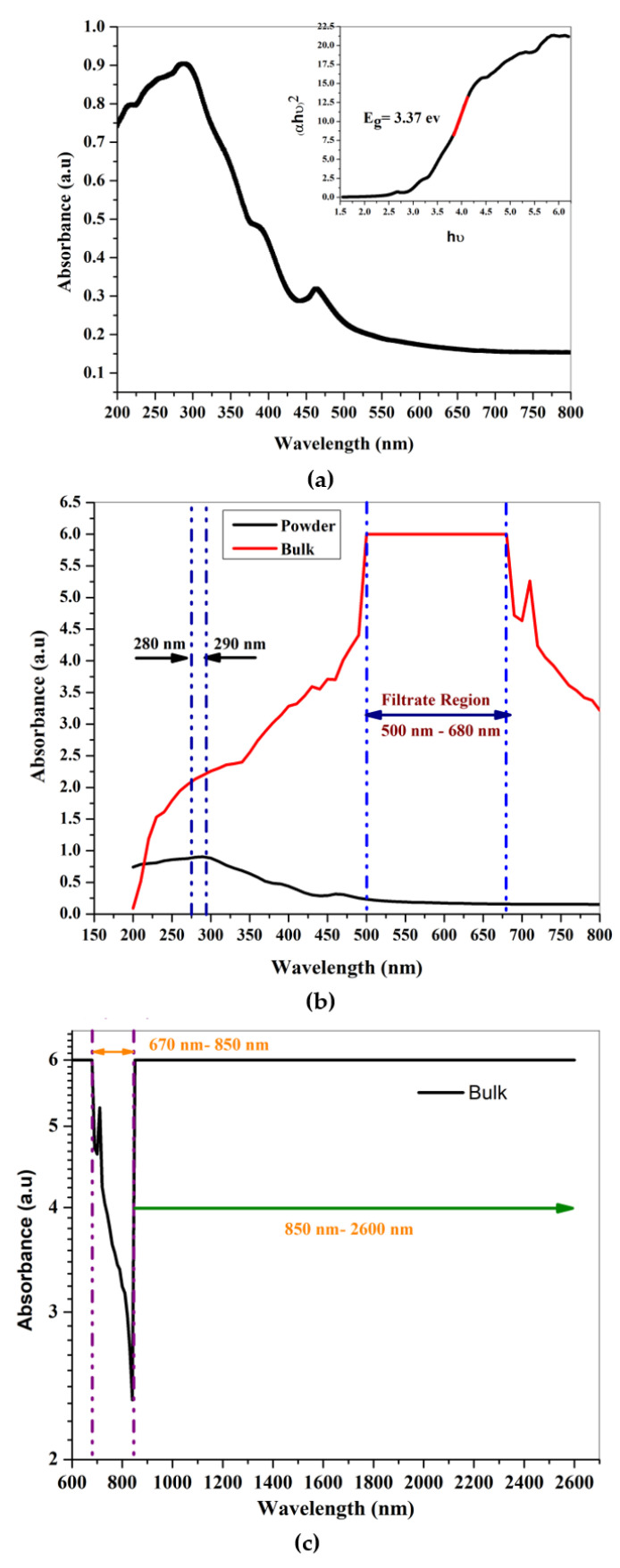
UV visible NIR spectra of (**a**) gahnite ferroan composite in powder form; (**b**) gahnite ferroan composite in powder and bulk form for comparative study and (**c**) gahnite ferroan composite of bulk sample beyond 600 nm wavelength.

The FT-IR spectrum of a prepared gahnite ferroan powder sample is shown in Figure 5. The most useful infrared region lies between 650 cm^−1^ to 4000 cm^−1^ since this radiation region induces the vibrational excitation of the functional groups. Above 3300 cm^−1^, there is no observation of any stretching or bending modes of vibration in the gahnite ferroan powder sample. Hence, Figure 5 denotes the wavenumber ranges from 550 cm^−1^ to 3300 cm^−1^. In the FTIR spectrum, sums of seven peaks are noticeable. The vibration modes of C-H bonds are observed at two positions of the wave number at 600 cm^−1^ and 640 cm^−1^. Another band appears at 950 cm^−1^, indicating the vibration modes of RCO-OH. There is observation of three stretching modes of vibrations at 670 cm^−1^, 1500 cm^−1^ and 1734 cm^−1^ assigned to C-Br, Ar C-C and C=O stretch modes, respectively. The modes of vibration at 2357 cm^−1^ indicate a P-H phosphine group. After diluting the materials in distilled water, the aqueous solution exhibits an alkaline pH value of 8.92. The optical absorption spectrum of gahnite ferroan is depicted in Figure 6a,b in a broad spectral range of 200 nm to 800 nm. The UV-visible spectrum of the prepared gahnite ferroan powder sample discloses two distinct absorption peaks at 288 nm and 363 nm. Absorption peaks arise due to band transition from the valence band to the conduction band. The direct band gap energy is determined by interpreting the plot between (αhν)^2^ and (hν). The calculated direct band gap energy of gahnite ferroan is 3.37 eV. Figure 6b depicts the comparative spectral study between the powder sample and the bulk sample. In powder form, the filtrate region is under ultraviolet wavelengths of light (280 nm–290 nm). In the case of the bulk form, the filtrate range (500 nm–680 nm) shifts toward the visible region with a prominent flat line of complete absorption. Beyond 850 nm to 2600 nm wavelengths, light is absorbed completely. As the bulk sample is processed using high-temperature sintering processes, grain growth occurs. A higher grain size reduces the optical band gap, for which both large grain and grain boundary are sensitive to absorb the particular wavelengths of light (500–680 nm).

After leaching, the produced gahnite ferroan is free from impurities and is not detected under the instrumentation limit. So, to use the powder in a final product, it may be coated over a substrate or directly made into a bulk form (pellet) to characterize its electrical properties. The bulk form of the sample was made directly from gahnite ferroan powder by using a hydraulic pallet press (Kimaya Engineers, Thane, Maharashtra, India) at 8 tons of pressure, for a standard time of 5 min. The thickness and diameter of the pellet was 2 mm and 13 mm, respectively.

**Figure 7:**
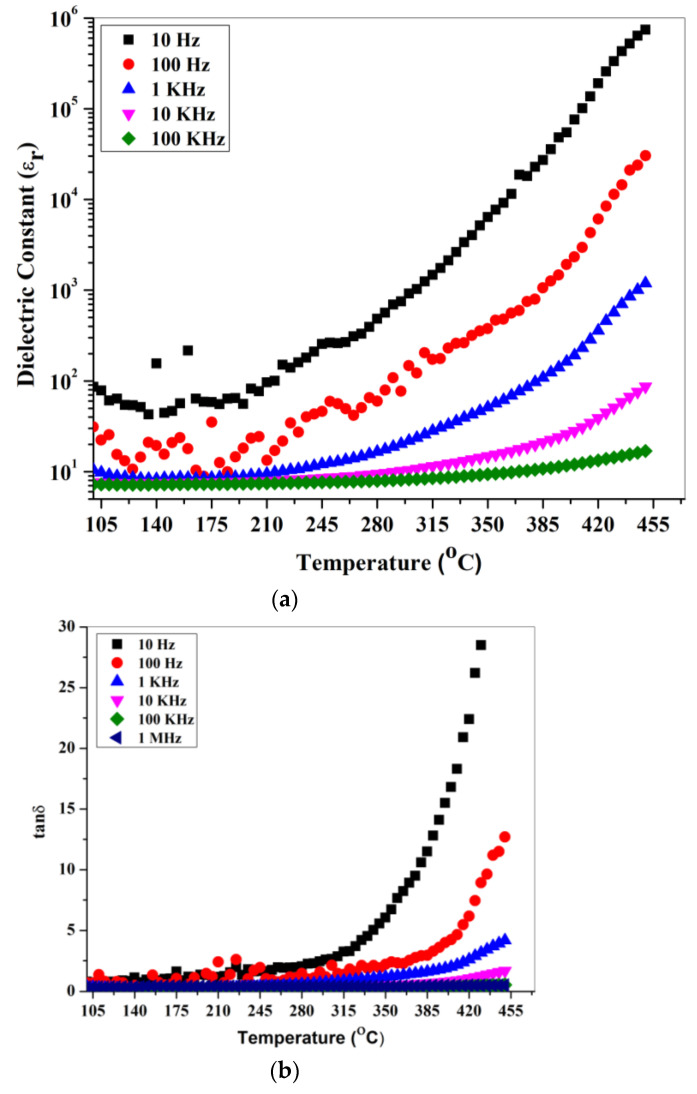
Temperature-dependent (**a**), dielectric permittivity and (**b**) dielectric loss of bulk gahnite ferroan composite, carried out at different frequencies.

The dielectric and electrical properties were analyzed by recording the AC parameters such as capacitance (C), loss tangent (tanδ), impedance (Z), and phase (θ) using an LCR meter (NF model ZM2376, Japan). The LCR meter analysis of the dielectric materials is based on the principle of the electrical circuit (RC circuit) modeling of dielectric materials. Each dielectric can be polarized by application of an external electrical field and has capacitance, C=ε0εrAd ($\varepsilon_{0}$: permittivity of free space, $\varepsilon_{r}$: relative permittivity/dielectric constant of the dielectric material taken, A: cross-sectional area of the specimen, d: the thickness of the specimen). For the electrical measurement of the gahnite ferroan, the pellets of 2 mm thickness were silver pasted and made conductive. The two opposite surfaces of the pellet, which are silver pasted, act as electrodes. The pellet was placed in a furnace using a sample holder which was connected to the LCR meter. Then, the AC parameters were recorded in a frequency range of 10 Hz–100 KHz by varying the temperature ranges from 75 °C to 450 °C. The LCR meter had a maximum six-digit resolution and a basic accuracy of 0.08%. Figure 7a,b depict the temperature-dependent variation in dielectric permittivity and dielectric loss of the prepared bulk sample at different frequency variations.

Some anomalies are observed at lower-end temperature values (~250 °C), which may be due to moisture and structural instability. Magnitude and dispersion in both dielectric permittivity and dielectric loss are increased with a rise in temperature (above 250 °C) up to the instrument upper limit (450 °C). This is due to a high-temperature sintering process. The dielectric is either perfect or imperfect. A perfect dielectric refers a good insulator, but in the case of an imperfect dielectric, the dielectric permittivity attains both real and imaginary functional parts. The real and imaginary parts act oppositely to each other. The real part is denoted as the dielectric constant. Elevation in temperature increases the dielectric constant value, whilst the imaginary part decreases it. Thus, it reveals the electric dipole information [32,33].

**Figure 8:**
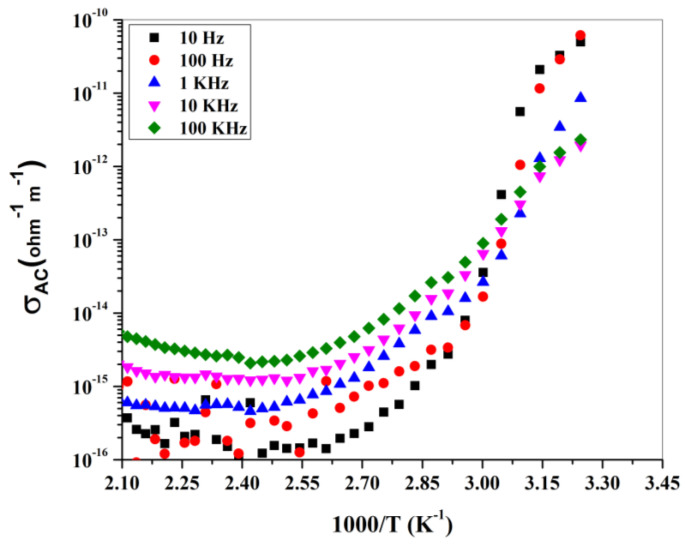
Arrhenius plot (ac conductivity study) of gahnite ferroan composite at different frequencies.

The case of a dielectric with a small semiconducting effect (imperfect dielectric) occurs due to charge carriers such as electrons, but in the case of gahnite ferroan material (powder form), it possesses a band gap of ~3.37 eV. The increase in dielectric constant at a higher degree of temperature reveals the presence of thermally activated charge carriers, whereas the imaginary part is referred to as the loss component of the material. The nature of the curve for both the dielectric constant and dielectric loss are similar to each other. The Arrhenius ac conductivity (logσ vs. 1000⁄(T) (K^−1^) study is depicted in Figure 8 at different frequency variations. The dispersion of conductivity occurs at high to low frequency values, with low 1000⁄(T) (K^−1^) values merged to a point and the order changed after that. The increase in the ac conductivity at a higher temperature denotes the negative temperature coefficient resistance (NTCR) behavior. The most common industrial applications of NTCR thermistors are found in modern electronic circuits [34]. The NTCR behavior enables the sample to be used for high-temperature sensing applications [35,36].

## 5. Conclusions

In conclusion, the gahnite ferroan has been derived from a low value-added material, i.e., from fly ash, thus indicating the conversion of waste to a value-added product. The gahnite ferroan nano composite has been successfully synthesized by chemical techniques followed by a solid-state reaction route, to a high-purity material confirmed by JCPDS # 85-1582. Surface morphology, structural appearance, texture and chemical composition of the samples were visualized using scanning electron microscopy in conjunction with an EDS system. The optical properties of the materials vary surprisingly, from nanocrystalline powder to the bulk form size of the material. The nano powder obstructs UV light, whereas the bulk form of the sample acts as a band pass filter for UV light. Hence, the same composition of gahnite ferroan composite may be used as a visible light filter or a UV light filter depending on the size of the product. In the case of dielectrics, a small semiconducting effect occurs due to charge carriers, such as electrons, but the gahnite ferroan material possesses a band gap of ~3.37 eV. An Arrhenius ac conductivity study of the bulk gahnite ferroan sample at various frequency ranges shows NTCR behavior, which illustrates the high-temperature sensing applications of these materials.

## Figures and Tables

**Table 1:** Quantification of various compounds present in the fly ash.

Compound	Quantification (wt%)
SiO_2_	57.13
Al_2_O_3_	34.24
CaO	2.84
MgO	0.91
Fe_2_O_3_	2.78
K_2_O	0.65
TiO_2_	0.91

## Data Availability

Not applicable.
